# Fingertip-Coupled Spindle Signaling Does Not Contribute to Reduce Postural Sway Under Light Touch

**DOI:** 10.3389/fphys.2019.01072

**Published:** 2019-08-22

**Authors:** Cristiano Rocha Silva, Fernando Henrique Magalhães, André Fabio Kohn

**Affiliations:** ^1^Biomedical Engineering Laboratory, Universidade de São Paulo, EPUSP, São Paulo, Brazil; ^2^Neuroscience Program, Universidade de São Paulo, São Paulo, Brazil; ^3^School of Arts, Sciences and Humanities, Universidade de São Paulo, EACH-USP, São Paulo, Brazil

**Keywords:** postural control, center of pressure, muscle spindles, fingertip touch, balance, haptic information

## Abstract

The details of how light touch (LT) of a stable surface reduces postural sway are still not well known. We hypothesized that removal of feedback provided by muscle afferents of the touching fingertip would increase postural sway in standing subjects. Eleven participants stood upright on a force plate with eyes closed and on an unstable surface. The experimental conditions involved two different finger positions: with partial muscle afferents (PMA), which includes sensory information from the fingertip flexor muscles, and no muscle afferents (NMA), without information from either fingertip flexor or extensor muscles. In the control condition, the participants kept the same posture, but with no finger touch (NT). Postural sway in both anteroposterior (AP) and mediolateral (ML) axes were recorded. Results showed that LT decreased all sway quantifiers as compared with the NT condition. The withdrawal of information from the touch finger muscle afferents (NMA condition) did not increase postural sway. Actually, there was a small, albeit statistically significant, decrease in the variability of center of pressure displacement in the AP direction. These results indicate that in some cases, muscle afferent input may either not contribute or even worsen the overall quality of sensory feedback from a given body segment, leading to no improvement or even a slightly decreased performance of the motor control system (evaluated by means of levels of postural sway in the present investigation). The results suggest that non-spindle fingertip afferents provide the bulk of the sensory feedback associated with the fingertip that is touching a ground-referenced object during quiet standing under LT.

## Introduction

The roles of sensory and motor interactions in the control of human postural sway have been largely explored. Sensory feedback mechanisms (mainly vision, somatosensory, and vestibular) are integrated to provide the central nervous system (CNS) with relevant information about body position and movement (Kouzaki and Masani, 2008; Ishigaki et al., 2016). In particular, the somatosensory system provides feedback signals associated with the location and motion of body segments, contact with external objects and postural orientation (Chen and Tsai, 2015). In this vein, lightly touching an external rigid surface has been demonstrated to reduce postural sway.

The “light touch (LT)” paradigm has been studied with different approaches. By far, the most studied LT condition has been when a fingertip is kept touching a fixed object (called “active touch”), thereby providing additional sensory cues as to the gravity force and the associated body sway. In this vein, experiments from different research groups demonstrated a markedly diminished postural sway in tandem (Lackner et al., 1999), semi-tandem (Baccini et al., 2007), single-leg (Bonfim et al., 2008), and bipedal (Franzén et al., 2012) stances. Within the scope of this fingertip LT, the putative mechanisms behind the effects of LT on postural sway have been associated with proprioceptive feedback from finger and hand muscles as well as from fingertip cutaneous mechanoreceptors (Holden et al., 1994; Jeka and Lackner, 1994; Clapp and Wing, 1999; Rabin et al., 2008; Baldan et al., 2014; Martinelli et al., 2015; Moraes et al., 2018).

Other LT conditions have also been studied, involving passive tactile inputs at different parts of the body (e.g., trunk, head, or limbs). These “passive LT” experiments also showed reduced postural sway, possibly without the influence of muscle spindle feedback (Rogers et al., 2001; Menz et al., 2006) and without the subject having a task of keeping his finger touching a given object (“active touch”). The results showed that the effects of such a “passive touch” on postural sway were dependent on the body part being touched (e.g., on the shoulders or on the legs). Other studies reported that even when the touch was applied to an object that was not fixed to the ground, the inertial forces could provide enough information of body position with respect to gravity to reduce postural sway (Krishnamoorthy et al., 2002). The latter group of researchers also compared the traditional LT with a condition where a clip pressed the tip of the finger (this led to less postural sway than without the clip). However, these variations associated with the effects of LT on postural control as well as the comparison between their results cannot be analyzed in a straightforward way, as there are many differences at the level of sensory receptors as well as at the systems level.

Therefore, the improvements due to LT may arise from different sets of sensory receptors, located at different parts of the body. In each case, interesting physiological questions arise at the different levels of complexity, from the microscale to the macroscale levels. In the microscale level, interesting questions arise as to the characteristics and the specific responses of the set of receptors activated by the LT. At a mesoscale level, interesting questions arise as to how the afferent inflows to the central nervous system interact/influence ongoing neural activity from spinal cord and brain neuronal networks that receive inputs from other somatosensory inputs (e.g., from the foot soles) as well as from the vestibular and visual systems. Finally, in a wider scale, quite complex questions arise on how these several neural activities integrate smoothly to influence the motor commands that keep the subject in quiet stance. The vast literature on LT has approached the studies more frequently from a black-box/systems level, without focusing on mechanisms at the receptor or neuronal levels, as done in the present research.

Studies of neural signals generated by human individual cutaneous or spindle afferents in different conditions are scarce due to the technical challenges of recording single afferent activity during the execution of specific sensorimotor tasks. While microneurography studies of fingertip cutaneous receptors have been reported by a few research groups in the world (Birznieks et al., 2009; Johansson and Flanagan, 2009), the concomitant study of both cutaneous and muscle spindle receptors has been much less frequent (Burke et al., 1988; Grill and Hallett, 1995). For example, only recently have single cutaneous and Ia muscle spindle afferent activities been studied with respect to movement of the human ankle. Ribot-Ciscar et al. (2013) were able to show that a few spindle receptors were responsive to added mechanical noise while cutaneous receptors were not affected by the added noise while signaling small movements of the ankle. Certainly, further studies are needed of the concomitant neural activities of cutaneous and spindle Ia and II afferents for a better understanding of their integrated physiological contributions.

To the best of our knowledge, the specific contributions of proprioceptive receptors linked to the fingertip for postural sway stabilization with LT have not been explored. Although there have been many empirical studies investigating how LT modifies postural control in individuals with a variety of sensory and motor impairments (see review in Baldan et al., 2014), no research has proposed to evaluate the manipulation of proprioceptive receptors of the hand and/or fingers during postural tasks with LT. In this paper, we hypothesized that the withdrawal of feedback from muscle Ia afferents from the touch finger, i.e., sensory information from muscle spindles, would increase postural sway as compared to a standard condition which includes both proprioceptive and cutaneous feedback.

To address this hypothesis, the current study employed the non-invasive methods proposed by Gandevia and McCloskey (1976) and Gandevia et al. (1983) in order to change the muscle afferent inflow from a fingertip during a LT postural task. These authors described an anatomical/biomechanical peculiarity that allows the hand to be positioned so that the flexor and extensor muscles of the terminal phalanx of the middle finger are held at lengths inappropriate for action and hence are effectively disengaged from the joint (in other words, the distal phalanx is loose and cannot neither be flexed or extended). This biomechanically constrained position is achieved when all fingers are extended except the middle finger, which is flexed 90° at the proximal interphalangeal joint (see Methods for details). When positioned this way, only joint and cutaneous mechanisms subserve position sense at the terminal phalanx (i.e., muscle afference from the fingertip is withdrawn). Additionally, the hand can be easily repositioned so that only the flexor (but not the extensor) of the terminal joint of the middle finger is engaged, which is achieved by flexing the index, ring, and little fingers so as to align them with the middle finger (Gandevia et al., 1983). These specific biomechanical schemes allowed Gandevia and collaborators to evaluate, from a perceptual point of view, the proprioceptive acuity of the terminal joint of the middle finger when no muscular afferents could contribute, when afferents of the flexor but not the extensor could contribute, or when afferents from both muscles were available. The authors assessed the participants’ ability to detect flexion and extension movements of the terminal phalanx of the middle finger by asking them to state their perception of the direction of movement and final position of the distal phalanx. In these studies (Gandevia and McCloskey, 1976; Gandevia et al., 1983), the scores for answers regarding the finger movements indicated that the presence of both agonist and antagonist muscle receptors led to superior proprioceptive acuity in comparison to that achieved when only the receptors of one of the muscle groups was available. Additionally, by nerve blocking cutaneous and joint receptors, the authors evidenced that the availability of muscle, joint, and cutaneous receptors is needed to achieve the full perceptual acuity of the middle finger, as a significantly poorer performance was observed when only joint and cutaneous receptors (or when only intramuscular receptors) were available.

Therefore, to unravel the specific contribution of the fingertip muscle afferents of the middle finger to the performance of LT postural tasks, we employed the methods briefly described above, as proposed by Gandevia et al. (1983), so as to assess the performance of the postural control system when the afferent inflow from the finger muscle sensors was removed. We hypothesized that removal of muscle afferent feedback from the fingertip touching a fixed object would increase postural sway. Thus, the purpose of the present study was to investigate the effect of the withdrawal of sensory inflow from muscle afferents of the middle fingertip on postural sway quantifiers. Preliminary results involving a somewhat different setup were recently published as a conference paper (Silva et al., 2019).

## Materials and Methods

### Participants

A group of 11 right-handed subjects (5 males, 6 females, age 29.1 ± 3.4 years, weight 67.7 ± 2.1 kg, height 1.7 ± 0.04 m, mean ± SD) were sampled by a non-probability (convenience) sampling, having volunteered to participate in this study. Handedness information was obtained by asking the participants which hand they preferred to use for writing and throwing a ball (McManus et al., 1999). The participants always performed the touch tasks described below with their right, dominant hand. All participants were healthy and physically active, with no known musculoskeletal injuries or neurological disorders that could have influenced their balance performance. The experiments were conducted according to the Declaration of Helsinki and all procedures were approved by the Human Ethics Committee of the Institute of Psychology at the University of São Paulo. Each participant signed an informed consent document prior to the experimental sessions.

The adequacy of the sample size in the present study was supported by computations based on the G*Power 3.1.7 software (Faul et al., 2007). A conservative estimation of the appropriate sample size was performed before the study based on data from pilot experiments and from a previous investigation regarding postural sway responses to light forces applied to the fingertip (Magalhaes and Kohn, 2011). The standard deviation relative to the mean was taken as 42% [from measurements with the largest standard deviations in the previous study, center of pressure (COP) Area]. The associated effect size was adopted as 0.3 (*η*^2^ values). Assuming a repeated measures approach, with four experimental conditions, with the power set at 0.8 (Cohen, 1988) and an alpha value of 0.05, the total sample size estimated in the present study resulted equal to 11.

### Center of Pressure and Fingertip Contact Forces

**Figure 1:**
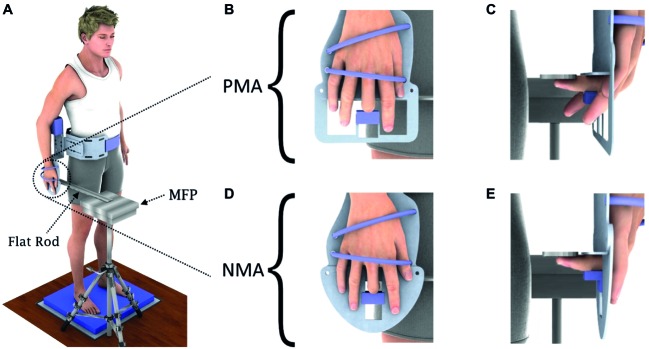
Representation of the experimental setup. **(A)** Depiction of participant standing quietly over the foam pad, with the mechanical apparatus fixed to his/her body and showing the flat rod used as an extension of the mini-force platform (MFP), which served as the touching surface for the participants’ fingertip. **(B,C)** Condition PMA, with the extensor muscle of the terminal joint of the middle finger disengaged. **(D,E)** Condition NMA, with both flexor and extensor muscles of the terminal joint of the middle finger effectively disengaged.

A force platform (OR6-7-1000, AMTI Inc., Watertown, MA, USA) was used to assess the ground reaction forces (GRFs) and COP during the postural tasks. The horizontal and vertical fingertip contact forces were measured with a 15.24 cm by 15.24 cm mini-force platform (MFP, see Figure 1A) (HE6X6, AMTI Inc., Watertown, MA, USA). The MFP was fixed over a tripod so that the height, position, and orientation of the touch surface could be adjusted to accommodate for differences in the participants’ heights. One of the tips of a flat and rigid aluminum rod (see Figure 1A) was glued to the MFP and the other tip served as the contact structure for the pad of the middle finger. To assure that fingertip forces would not provide significant mechanical support, a custom-written software (NetForce, AMTI Inc., Watertown, MA, USA) was used to measure the forces applied to the MFP during the tests. If, at any time, more than 1 N was applied by the participant’s finger, the trial was rejected and repeated.

### Foam Pad

A foam rubber pad (Airex balance pad, Alcan-Airex AG, Sins, Swiss) with 49.5 cm length, 40.5 cm width, 66.5 cm height, 0.013 m^3^ volume, and 58.38 kg/m^3^ density was positioned over the force platform during all trials in order to challenge the postural control system. The unstable support surfaces reduce the sensory feedback and the effectiveness of corrective ankle torque (Patel et al., 2008; Hatton et al., 2009). In the current study, the rationale behind this choice was that the unstable support surface challenges the sensorimotor system and accentuates the need for extra sources of sensory information (as those from the finger touch) other than those from the foot level.

### Mechanical Apparatus to Investigate the Contribution of Middle Finger Muscle Sense

Based on the methods proposed by Gandevia and McCloskey (1976) and Gandevia et al. (1983), a mechanical apparatus was developed to keep the participants’ right hand in specific postures in which the contribution of the muscular receptors of the middle finger could be investigated. Additionally, based on immobilization techniques proposed by Rabin et al. (2008), the mechanical apparatus also reduced the degrees of freedom of the arm by immobilizing the right upper arm, forearm, and hand with respect to the trunk. This condition eliminated any motor contribution of the arm, thereby keeping arm proprioception relatively constant as the joint positions were unchanged during the experiments.

The apparatus was composed of an aluminum structure attached to the waist of the subject. The lower part of the aluminum structure had two different interchangeable plates. The first plate (see Figures 1B,C) was designed to keep the index, ring, and little fingers free, so that the terminal joint of the middle finger could be flexed but not extended by voluntary effort and hence the flexor muscle of the middle finger was kept engaged (denoted “with partial muscle afferents,” PMA). The papers by the group of Gandevia and McCloskey (1976) and Gandevia et al. (1983) cited above provide the experimental and conceptual support for the PMA condition. The second plate (see Figures 1D,E) was designed to keep index, ring, and little fingers extended, while the middle finger alone was held flexed 90° at the proximal interphalangeal joint, so as to withdraw the sensory information from both flexor and extensor muscles of the middle finger (denoted “with no muscle afferents,” NMA), as explained by Gandevia and McCloskey (1976) and Gandevia et al. (1983). In both positions, the dorsal aspect of the second phalanx of the third finger was in contact with a small plastic piece that kept this phalanx immobilized. The distal phalanx was kept approximately aligned with the second phalanx (i.e., there was an approximately 180° angle at the distal interphalangeal joint) as the fingertip was touching the flat rod. The hand was fastened firmly to each plate, hence avoiding movement of the hand and fingers with respect to the plate. Summarizing, in position NMA, no feedback must have arisen from intramuscular receptors of either flexor or extensor muscles that respond to movement of the distal phalange of the third finger. On the other hand, in position PMA, intramuscular muscle receptors from the flexors of the distal phalange were potentially able to respond to angular changes of the most distal interphalangeal joint. In neither condition did the small plastic piece used to fix the middle finger cause any pain or discomfort to the subject and neither gave a differential tactile feedback to the subject. Due to the fastening of the hand to each plate, specific cutaneous or joint feedback due to postural oscillations was non-existent from the hand and fingers 1, 2, 4, and 5.

### Experimental Procedures

A schematic illustration of the experimental setup is shown in Figure 1. The mechanical apparatus was properly attached to the participants’ body (see Figure 1A) so that the corresponding right arm was positioned by the body side with no muscles activated (i.e., in a relaxed state). There were no specific instructions given to the subjects besides staying at ease. No attention was necessary to keep the finger in touch with the MFP since the fingertip was kept in touch with the aluminum flat rod (see Figure 1A) by a double-sided adhesive tape, providing a fixed contact surface for the middle finger during all trials.

Participants naturally hung their left arm down, while the right shoulder was slightly abducted to allow the correct attachment of the apparatus in this condition. The right arm and right forearm remained aligned with the trunk during the tests, with the elbow at approximately 170° (see Figure 1). During the tests, the metacarpal region was maintained fixed by two bands to a metal plate (Figures 1B,D) which was part of the apparatus. For both NMA and PMA conditions, the thumb remained fully extended while the middle finger’s (used for touching) medial and distal phalanges were aligned and remained at approximately 90° with respect to the proximal phalange, which was aligned with the third metacarpus. The joint position of the index, ring, and little fingers changed between the NMA and PMA positions: in the NMA position (see Figures 1D,E), the metacarpophalangeal, proximal interphalangeal, and distal interphalangeal joints of the index, ring, and little fingers were fully extended, while in the PMA position (see Figures 1B,C), the index, ring, and little fingers were kept free. During the experiments, participants were required to select a comfortable position and to keep a quiet barefoot stance on a foam pad (over the force platform), with feet apart at approximately shoulder width and with the eyes closed. As the subject could not move the arm and the hand, the MFP was positioned by the investigator so that the fingertip touched the MFP positioned above the right middle finger. Additional bands were used to immobilize the wrist and elbow joints. Moreover, it is important to emphasize that the sensory feedback from other parts of the hand and also from the fingers was very much equalized between the PMA and NMA conditions under the setup shown in Figure 1, except for that coming from the last phalanx of the middle finger. The hand was fixed with two straps to an aluminum plate so that no movement could occur of hand and fingers (except for the third finger’s distal phalanx) due to postural sway. The plastic block pressed the dorsum of the third finger equally between PMA and NMA conditions and it is quite improbable that it could be a source of differential cue signals between the two conditions.

The MFP vertical force was minimized by the investigator by adjusting the tripod height so that the vertical force did not exceed ±1 N. After finding the proper position of the MFP, the tripod leg positions were marked on the ground and its height was also annotated. Such information was used to standardize the touch conditions within each experiment. Following these procedures, the position of the subject’s feet on the foam pad was marked with adhesive tape to ensure that the same position relative to the touching apparatus was kept along the entire experiment. The participants had no previous knowledge about the experimental design and hypotheses, and they were not given feedback about their postural performance. That is, the participants were not asked to do anything other than stand upright quietly in bipedal stance.

In addition to the two finger positions (PMA and NMA) commented above, two touch conditions were used: (1) LT, in which the middle finger touched the flat rod and hence the MFP; and (2) NT, no contact with the flat rod, but with the same arm, hand, and finger positions as in PMA and NMA (the MFP with the attached flat rod was removed from the vicinity of the subject, remembering that the subject performed all the experiments with closed eyes). Thus, each participant was tested under four experimental conditions: PMA-NT, PMA-LT, NMA-NT, and NMA-LT. Five trials were performed for each condition (presented in a randomized order), each lasting 100 s, and a resting period of ~120 s between the trials was allowed to avoid fatigue (subject sat in a comfortable armchair placed next to the force platform). To avoid transient adaptations, data recording started after 10 s from the beginning of each trial, resulting in a trial of 90 s. The experimental session lasted approximately 1 h.

### Data Analysis

All data were acquired with a sampling frequency of 100 Hz using a 16-bit A/D converter (Optotrak Data Acquisition Unit, Northern Digital Inc., Ontario, CA) controlled by a NDI First Principles software (Northern Digital Inc., Ontario, CA). The forces and moments measured by the force plate were used to compute the two components of COP: in the anterior-posterior axis (AP) and the mediolateral axis (ML), indicated as COPap and COPml, respectively. The COP signals in both directions were passed through a low-pass filter of 10 Hz using a fourth-order Butterworth filter. The mean was subtracted from each time series and the root mean square (RMS) and mean sway velocity (MSV) of the COP data were computed for each axis (i.e., AP and ML). The COP MSV was calculated by dividing the total COP displacement by the total time interval. Time domain COP measures (RMS and MSV) were computed for each trial, and the mean of five trials for each experimental condition was calculated for each subject.

The power spectral densities (PSDs) of the COP data, for the AP and ML axes, were estimated in each experimental condition. The average power spectrum obtained in each condition from all 11 participants was calculated. The PSDs were estimated by the Welch periodogram method of the detrended data with 2,000 samples per periodogram, resulting in a resolution of 0.05 Hz. A Hann data window was used with subtraction of the best linear regression and an overlap of 1,000 samples (50% overlap). The area under the PSD was evaluated for each trial at two frequency bands: “low frequencies” (LF, 0.05–0.5 Hz) and “high frequencies” (HF, 0.5–2.0 Hz) (Magalhaes and Kohn, 2011). The upper limit of the high-frequency range was chosen because 99% of the total power of the COP signal during standing has been reported to be below 2 Hz (Mezzarane and Kohn, 2008).

To assess the influence of a putative mechanical support by the fingertip on body sway, the absolute values of the vertical and horizontal fingertip forces were compared between NMA and PMA conditions. Additionally, the standard deviations (SDs) of the horizontal GRFs in the AP direction (during LT and NT conditions) were calculated and compared with the SDs of the horizontal fingertip forces in the AP direction (Kouzaki and Masani, 2008).

All data analyses employed customized programs written in Matlab R2015a (Mathworks, Inc., Natick, MA, USA).

### Statistical Analysis

Normality of the data was tested using the Kolmogorov-Smirnov method (*p* < 0.05), which indicated that all dependent variables were normally distributed. Therefore, parametric tests were used for comparisons. The COP parameters (RMS, MSV, LF, and HF) were compared with a two-way ANOVA with repeated measures, in order to detect possible differences and interactions between finger position (PMA vs. NMA) and fingertip contact (NT vs. LT) conditions. All analyses were performed using the statistical package SPSS 22.0 for Windows (SPSS, Inc., Chicago, IL, USA). An alpha level of 0.05 was chosen for all initial statistical comparisons, with a least significance difference (LSD) *post hoc*^1^ comparison performed when necessary (Field, 2017). A two-tailed paired *t*-test was used to compare the absolute values of the fingertip forces between NMA and PMA conditions (*α* was set at *p* < 0.05). All data are given as means ± standard error of the mean (M ± SEM) in the text and figures. Effect sizes (also known as “strength of association”) were expressed as partial eta-squared ($\eta_{p}^{2}$: proportion of variance that a variable explains that is not explained by other variables) and Cohen’s *d* (difference between groups in terms of standard deviation units). Cohen (1988) made some widely used suggestions about cut-off values: small ($\eta_{p}^{2}$ ≥ 0.01; *d* ≥ 0.2), medium ($\eta_{p}^{2}$ ≥ 0.06; *d* ≥ 0.5), and large ($\eta_{p}^{2}$ ≥ 0.14; *d* ≥ 0.8). Adjustments proposed by Cumming (2012) were applied to estimate unbiased Cohen’s *d* values, then the Hedges’ correction was conducted (Hedges, 1981).

## Results

**Figure 2:**
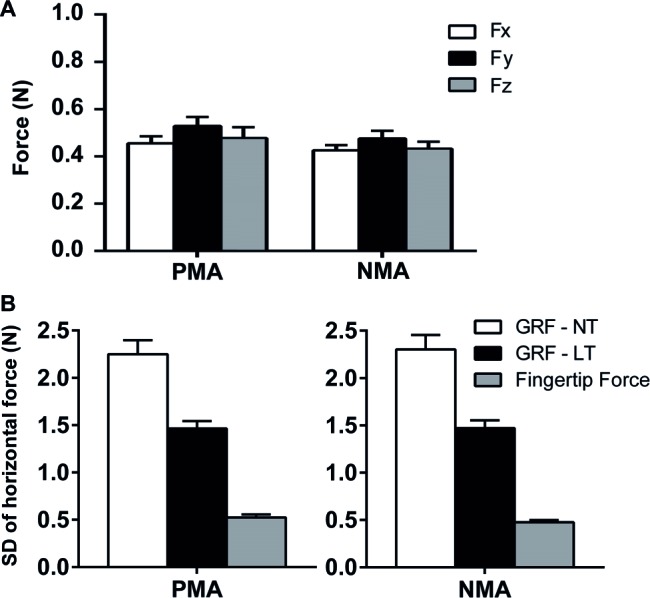
Fingertip and ground reaction forces (GRFs) measured across the experimental conditions. **(A)** Vertical (Fz) and horizontal (Fx and Fy) forces (M ± SEM) applied to the MFP during the LT conditions. There were no statistical differences for the force values between the PMA and NMA conditions (*p* > 0.05). **(B)** Shows the standard deviations (SDs) of the horizontal GRFs in the AP direction (during LT and NT conditions) and the SDs of the horizontal fingertip forces in the AP direction (PMA and NMA conditions are shown in left and right panels, respectively).

The accepted criterion for a haptic feedback to be considered a LT and not a mechanic support is that the vertical force (Fz) applied by the fingertip should not exceed 1 N (Holden et al., 1994; Jeka and Lackner, 1994). Careful inspection of individual data was performed to verify that this condition was satisfied overall, during the whole set of experiments. During LT conditions (i.e., during PMA and NMA finger positions), the mean forces in all three directions, Fx (anterior-posterior direction), Fy (mediolateral direction), and Fz (vertical direction), applied by the fingertip were below 1 N for all trials performed by the subjects. A paired-samples *t*-test was conducted to evaluate the fingertip force values between the PMA and NMA conditions. There was no statistically significant difference between the fingertip forces in the Fx (*t*_(54)_ = 0.46, *p* = 0.64, $\eta_{p}^{2}$ < 0.01, *d* = 0.08), Fy (*t*_(54)_ = 1.36, *p* = 0.052, $\eta_{p}^{2}$ = 0.03, *d* = 0.44), and Fz (*t*_(54)_ = 0.92, *p* = 0.36, $\eta_{p}^{2}$ = 0.01, *d* = 0.14) comparing the PMA and NMA conditions (see Figure 2A). Additionally, the SD of the horizontal GRF forces in the AP direction was considerably higher (PMA-NT = 2.25 ± 0.14 N; NMA-NT = 2.30 ± 0.15 N; PMA-LT = 1.46 ± 0.08 N; NMA-LT = 1.47 ± 0.08 N) than the fingertip forces in the AP direction (PMA-LT = 0.52 ± 0.03 N; NMA-LT = 0.47 ± 0.02 N) (Figure 2B), which suggests the LT effect was associated with sensory mechanisms rather than with a mechanical support (Kouzaki and Masani, 2008).

**Figure 3:**
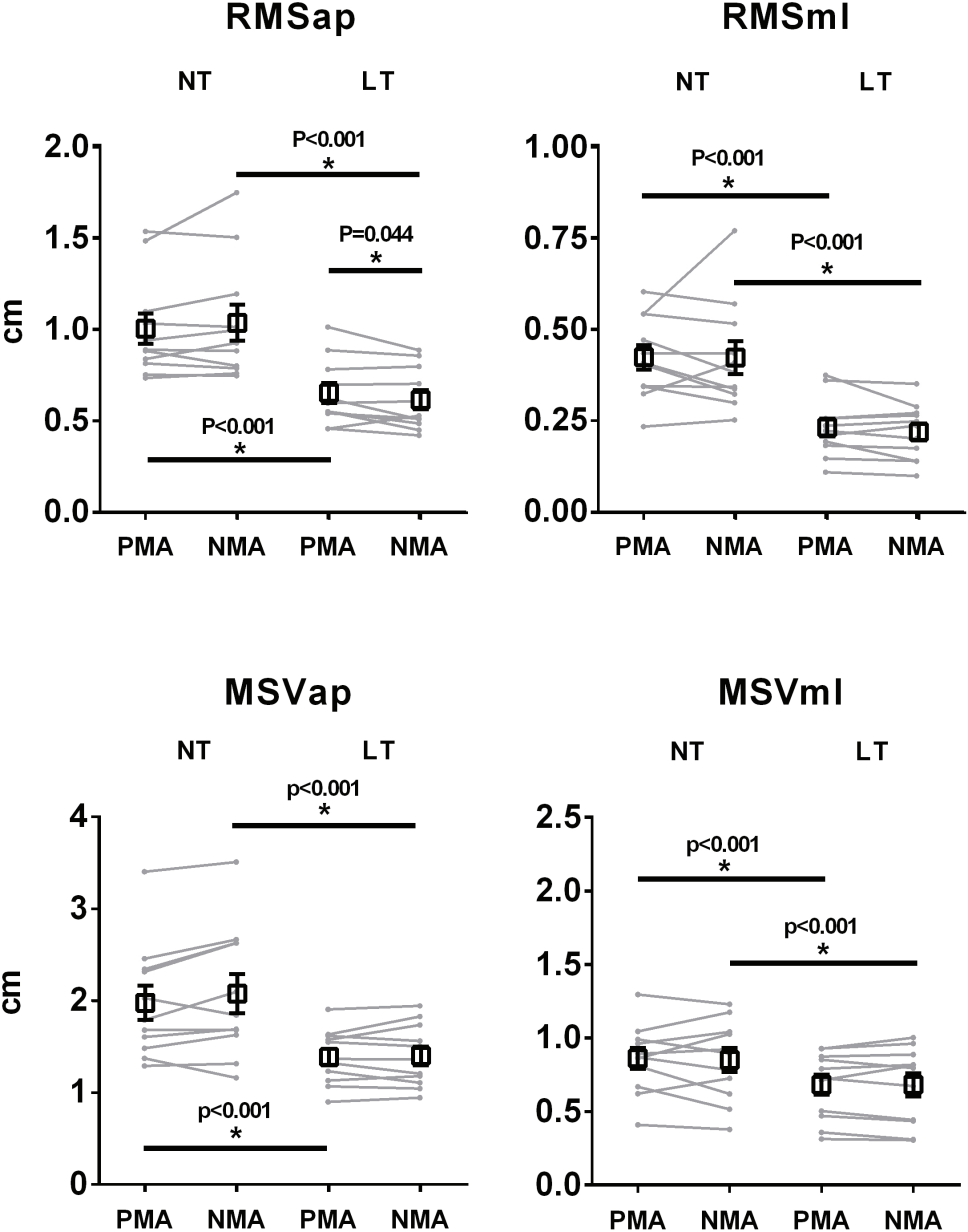
Averaged (squares) and individual (gray lines) RMS and mean sway velocity (MSV) measurements (M ± SEM) computed from the COP signals in both AP and ML directions. * indicates significant (*p* < 0.05) main effects for light touch, and significant interactions between light touch and finger position.

Figure 3 depicts the mean (black squares) and individual (gray lines) values of COP RMSap, RMSml, MSVap, and MSVml for PMA and NMA finger positions and for LT and NT conditions. There was a statistically significant main effect for fingertip contact for RMSap [*F*(1, 54) = 259.62, *p* < 0.001, $\eta_{p}^{2}$ = 0.82, *d* = 1.09], RMSml [*F*(1, 54) = 239.54, *p* < 0.001, $\eta_{p}^{2}$ = 0.81, *d* = 1.29], VMap [*F*(1, 54) = 102.08, *p* < 0.001, $\eta_{p}^{2}$ = 0.65, *d* = 0.86], and VMml [*F*(1, 54) = 49.47, *p* < 0.001, $\eta_{p}^{2}$ = 0.48, *d* = 0.51], indicating a decrease in postural sway during the LT condition as compared to the NT condition, regardless of finger position. There was a significant interaction effect between finger position (PMA vs. NMA) and fingertip contact (NT vs. LT) for RMSap (*F*(1, 54) = 5.21, *p* = 0.026, $\eta_{p}^{2}$ = 0.08, *d* = 0.21), with LSD *post hoc* comparisons indicating that there was a statistically significant decrease in RMSap for NMA as compared with PMA (*p* = 0.044) during the LT condition, but with no differences between PMA and NMA during the NT condition (*p* > 0.1). There were no significant differences between PMA and NMA conditions for RMSml, MSVap, and MSVml.

**Figure 4:**
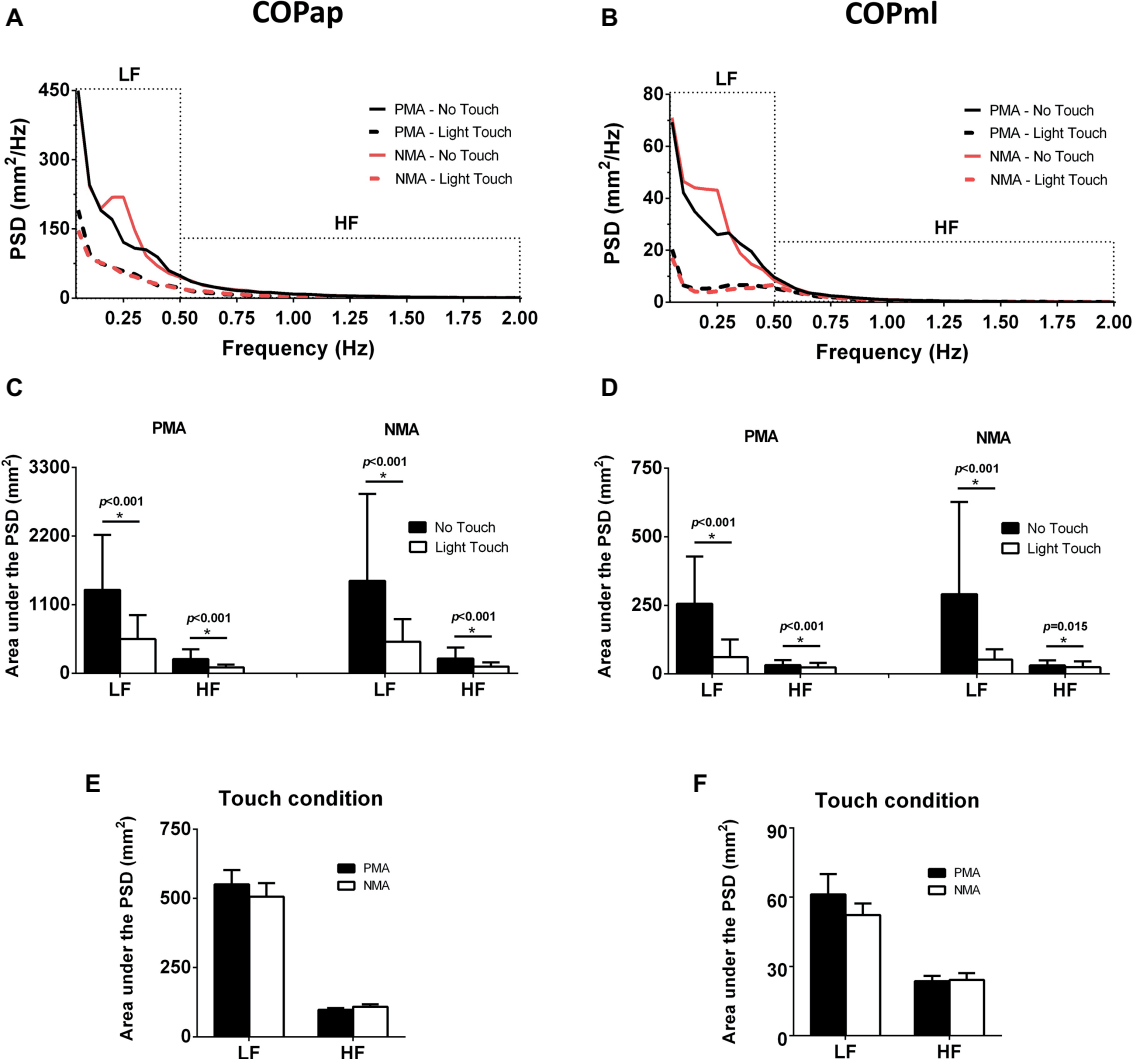
Frequency-domain analyses showing the power spectral densities (PSDs) of the COP signals in different conditions. **(A)** Mean power spectra (*n* = 11) of COPap. **(B)** Mean power spectra of COPml. **(C)** Bar plots show the areas under the COPap PSDs divided into low-frequency (LF, 0.05–0.5 Hz) and high-frequency (HF, 0.5–2 Hz) bands. **(D)** The same as in C, but for COPml. **(E)** Areas under the PSDs during LT in the PMA and MNA conditions measured from the COPap. **(F)** The same as in E, but for the COPml. * indicates significant differences (*p* < 0.05).

Figure 4 shows the frequency-domain analyses of the COP signals, with mean PSDs (see Figures 4A,B) and averaged LF and HF areas under the PSDs (see Figures 4C,D) for each condition. The area under the PSD of the COPap and COPml showed a statistically significant main effect for the touch condition at low and high frequencies: LFap [*F*(1, 54) = 60.74, *p* < 0.001, $\eta_{p}^{2}$ = 0.53, *d* = 0.75]; HFap [*F*(1, 54) = 59.90, *p* < 0.001, $\eta_{p}^{2}$ = 0.53, *d* = 0.77]; LFml [*F*(1, 54) = 73.74, *p* < 0.001, $\eta_{p}^{2}$ = 0.58, *d* = 0.95]; and HFml [*F*(1, 54) = 12.95, *p* = 0.001, $\eta_{p}^{2}$ = 0.19, *d* = 0.30]. No significant main effects of finger position (PMA and NMA) and no significant interactions between finger position and fingertip contact (NT vs. LT) were found, regardless of the direction of the COP signal (AP and ML). Although no significant differences were found between finger positions during the LT condition (shown in appropriate ordinates in Figures 4E,F), there was a decreasing trend from PMA to NMA in the LF band.

## Discussion

The purpose of the present study was to investigate whether the removal of muscular afferent feedback from the middle finger leads to an increase in postural sway during LT conditions. The rationale was that the removal of proprioceptive feedback from muscle afferents during the postural task with LT would worsen the performance of the postural control system. This would result in increased postural sway as compared to the condition with available information from muscle afferents.

The LT was able to decrease all the adopted COP quantifiers compared with the NT condition confirming previous data from the literature (Clapp and Wing, 1999; Baccini et al., 2007; Magalhaes and Kohn, 2011). However, the main finding of the present research was that the removal of feedback from a set of muscle afferents from the distal phalanx during LT (NMA) did not worsen the postural sway displacements. Actually, it even decreased the postural sway displacement variability (RMS in AP direction) by a small but statistically significant amount (see Figure 3). The results from the PSD frequency bands reinforce the deductions that can be made from the results of Figure 3. The areas under the COP PSD between the PMA and NMA in the HF bands (in both AP and ML directions) were similar and statistically indistinguishable (Figures 4E,F), which was consistent with the finding of no statistical differences in MSV in the COP analysis. The LF bands showed a decreasing trend (albeit not statistically significant) in power between finger positions (Figures 4E,F), whereas the RMS variable (in AP direction) was more sensitive to detect the differences between PMA and NMA, indicating a reduction in postural sway when the information from muscle afferents were removed.

The results presented above indicate that there was no worsening of postural sway parameters when the muscle spindles associated with the fingertip flexors and extensors were disengaged (i.e., not signaling changes in muscle length). These results suggest that the inclusion of spindle feedback (i.e., PMA condition) could not provide an overall improvement of the population afferent signals used by the postural control system to stabilize body sway. In other words, both the results that evidenced an enhanced stabilizing effect for the NMA condition as compared to the PMA condition and the results that showed no difference between NMA and PMA conditions contradict the initial hypothesis of the present study (i.e., that the removal of muscle afferent feedback would increase postural sway).

In the present study, there were no statistical differences between fingertip forces across the conditions, ruling out the possibility of a mechanical support effect in the results associated with the comparisons between PMA and NMA conditions (see Figure 2A). Moreover, the mean fingertip forces were actually slightly decreased in NMA as compared to PMA, which is exactly the opposite that would occur in the case of a stabilizing effect due to mechanical support, reinforcing that the postural sway reduction in the RMSap of the NMA as compared to PMA was probably due to sensory feedback mechanisms. A previous investigation by Kouzaki and Masani (2008) addressed whether a putative mechanical support could be the cause for LT-induced decreased postural sway. The authors showed that body sway was significantly smaller in the LT condition as compared to the NT condition, whereas LT effect was not significant under diminished finger sensory feedback (induced by tourniquet ischemia), despite the similar horizontal finger forces between conditions. In addition, they showed SDs of fingertip contact forces about five times lower as compared to horizontal GRFs. Similar to Kouzaki and Masani (2008) experiments, in the present study, the SDs of the horizontal fingertip forces in the AP direction were much lower than the horizontal GRFs in the AP direction (Figure 2B), thereby suggesting that the LT effect was associated with sensory mechanisms rather than with a mechanical support (Kouzaki and Masani, 2008). However, one must interpret these results with caution, as the sensory conditions used in the study of Kouzaki and Masani (2008) were indispensable to rule out the possibility of a mechanical support as the cause of LT-induced decreased body sway, which was not the aim of the present study.

Previous studies have suggested an important role for cutaneous and proprioceptive receptors in providing the CNS with information about the position of the finger during LT postural tasks, thereby leading to decreased body sway (Dickstein et al., 2001; Lackner et al., 2001; Krishnamoorthy et al., 2002; Rabin et al., 2008; Prado-Rico et al., 2018). Therefore, by canceling the sensory information sent by one of the finger muscles during the LT postural task, we expected to observe an increase in postural oscillations because the CNS would receive a less precise or less complete sensory feedback arising from the finger (Gandevia et al., 1983). The results of the present experiments are somewhat surprising as Gandevia and McCloskey (1976) and Gandevia et al. (1983) observed that the withdrawal of the muscular sensory information of the interphalangeal joints of the third finger of the hand diminished the perception of low amplitude angular displacements. These studies have shown that the contributions of all receptors (skin, joints, and muscles) are necessary for optimal accuracy of perception. However, it should be emphasized that afferent inflow below conscious detection may still provide important feedback for motor control. For example, perceptually subthreshold electrical stimulation of leg muscles has been shown to improve motor control at appropriate intensities of the subliminal electrical stimulation (Magalhaes and Kohn, 2012; Magalhães and Kohn, 2014). Therefore, information from perceptual experiments should be applied with some care when interpreting how the CNS processes sensory information to activate specific neuronal networks in the spinal cord and in the brain, e.g., related to motor control.

There are at least three different approaches to study the contributions of cutaneous and muscle receptors as sources of feedback to the human CNS: perceptual (e.g., in position or movement senses), by microneurography, and by their actions on the motor system. Perceptual investigations have suggested that the combined inputs from cutaneous and muscle receptors provide relevant position and movement feedback at different places of the body (Gandevia et al., 1983; Collins et al., 2005; Cordo et al., 2011), assuming the investigated joint is not at one of its two extreme positions. Such indications have been confirmed by microneurography studies (Burke et al., 1988; Grill and Hallett, 1995). The influence of cutaneous and muscle spindle sensory feedback on motor systems is probably more complex to understand, not only because it involves additional neural processing at the motor system level, but also because sensory feedback may be relevant for motor control even if it does not reach consciousness (Magalhaes and Kohn, 2012). The present experiments tried to dissect how relatively small subsets of cutaneous and muscle spindle receptors may influence a particular type of motor behavior (i.e., postural control). From the focused question that was posed in the present experiments, it seems that non-spindle mechanoreceptors at the fingertip were generating an accurate enough feedback signal so as to provide the motor control system (spinal cord and upper levels) with useful information to improve a motor action, i.e., reduce postural sway. At the same time, the present data suggest that when spindle feedback was included, it did not provide a clear enough afferent signal to be useful for the motor system. This could occur, for example, if the signal-to-noise ratios of the proprioceptive signals were small enough to cause a deterioration of the overall quality of sensory inflow used to control the motor action (resulting in increased postural sway as measured by some of the COP measurements).

The perception of movement is thought to be greater during an active movement than during a passive movement (Fuentes and Bastian, 2010; Cordo et al., 2011; Papetti et al., 2017). Previous studies have shown that muscle activation leads to the recruitment of the respective muscle spindles due to fusimotor activation (Burke et al., 1978), potentially improving the signaling of muscle length changes. Additionally, attention has also been shown to change muscle spindle sensitivity in humans due to modulation of fusimotor control (Hospod et al., 2007), and the role of attention during LT postural tasks has also been addressed (Riley et al., 1999; Vuillerme et al., 2006; Chen et al., 2017). In most of experiments in the literature using LT postural tasks, there was an active control of fingertip position by the subject on the contact reference surface and therefore the contribution of the fingertip-associated spindles (among many others associated with other phalanges and other segments in the hand and arm) must have been accurate enough for useful feedback (Lackner et al., 2000; Lackner and DiZio, 2005; Rabin et al., 2008). In our study, however, there was no direct instruction requiring the participants to flex or relax the fingertip that was touching the surface, as the only instruction given for the participants was “to keep a quiet, relaxed stance.” Additionally, there was an adhesive tape keeping the fingertip in contact with the reference surface and the contact between the touching surface and the fingertip was achieved by adjusting the height of the MFP (i.e., participants did not have to perform active movements or pay attention to guarantee that the fingertip remained touching the reference surface). Therefore, we can suppose that there was no (or very little) fingertip flexor muscle activation and that the attentional effort was minute in the experiments, both issues contributing to a probably absent fusimotor drive (Burke et al., 1979) and hence a poor transduction of the little changes in flexor muscle length that the postural sway must have caused at the distal interphalangeal joint in PMA condition. On the other hand, in position NMA, both flexor and extensor spindles were disengaged and no spindles responded to the small angle changes of the distal interphalangeal joint, and hence a relatively accurate non-spindle signaling must have acted without a parallel, imprecise or noisy, muscle afferent input.

Receptors in the interphalangeal joints are stimulated primarily at positions near to the maximum amplitude of the finger’s range of motion and are unable to detect the direction of movement or joint position at normal, intermediate amplitudes (Proske and Gandevia, 2009, 2012). In the present experiments, as the distal phalanx was in approximate continuity with the previous phalanx, and no force occurred that could lead to hyperextension of the distal phalanx, it is very likely that no joint receptors (or quite few) have been stimulated in either experimental condition (PMA and NMA) (Burke et al., 1988), suggesting that in the present study we could focus on the muscle spindles and the cutaneous receptors associated with the fingertip.

There are results from the literature that provide support to the above explanations of our results, with the caveat that they are related to conscious sensory processing. For example, Cordo et al. (2011) investigated the existence of redundant information generation by cutaneous and muscular receptors on position sensitivity of the finger, more specifically in the metacarpophalangeal joints. The authors observed that the proprioceptive information of the skin and the muscle spindle are not entirely redundant, with each set of information contributing in a different way to the sensitivity of the hand. The authors found that the sensation associated with the dynamic and static position of the fingers derives mainly from the cutaneous receptors, whereas the speed of movement derives from both the skin receptors and the muscle spindles. According to Clark et al. (1985), the more distal the joint, the smaller the contribution of the respective muscle spindle to proprioception sense seems to be. These data from the literature, taken together with the results of the present study, support the hypothesis of a greater importance of the tactile elements in providing sensory feedback from the more distal joints of the human fingers during LT.

The results of the present study may also be looked from a biomechanical perspective. During postural sway with LT, there will be time-varying changes in skin stretch and fingertip pressure (i.e., tangential and normal forces to the fingertip) that will reflect a distorted version of postural sway (due to viscoelastic nonhomogeneous and anisotropic properties of the involved biological structures). At the same time, there will be a time-varying rotation of the distal interphalangeal joint due to postural sway. The latter will lead to pulls and releases of the very long tendons of the flexor and extensor digitorum muscles of the distal phalanx (whose muscle belies are located in the forearm). The long tendons that need to pass through several joints until reaching the last phalanx (used in the LT condition) must lead to a weak/noisy and distorted mechanical version of phalanx rotation at the muscle spindles, and hence spindle output may be unreliable and/or noisy. Additionally, the muscles that flex or extend the fingers are multi-articular and each set of four tendons (e.g., for extension) of a given finger come from a single forearm muscle, creating ambiguity (noise) in the afferent inflow from muscle spindles as to which joint of the finger is being subjected to angular changes (Sturnieks et al., 2007; Cordo et al., 2011; Proske and Gandevia, 2012). These considerations are consistent with the conclusions from the experiments by Clark et al. (1985), i.e., that more distal joints contribute with less important muscle spindle feedback.

It is important to emphasize that the level of stimulation of skin receptors has not been explored in this study. The increase/decrease in the firing rates of all receptors was not assessed. We believe that the changes observed in COP quantifiers are capable of translating (indirectly) possible phenomena that may have occurred with the proprioceptive sensory receptors during the experiments. More invasive studies, using, for example, microneurography techniques (Birznieks et al., 2001, 2009; Ribot-Ciscar et al., 2013) would be able to shed further light on the specific contributions of the cutaneous and muscle receptors that provide sensory feedback during a LT protocol. Further research evaluating the behaviors of additional variables, such as the time course of the center of mass, surface electromyogram, and segmental/body angles, may contribute to a deeper understanding of how the withdrawal of proprioceptive sensory information influences postural control. The present experiments were performed with eyes closed standing on foam. The rationale was that the lack of visual information and the decreased accuracy of ankle proprioceptive signals (besides the decreased effectiveness of ankle torque in adjusting body position) would challenge the neuromuscular system by accentuating the need for other sensorial information (such as those from the cutaneous and muscle afferents from the finger), thereby increasing the probability of observing a putative effect of the muscle afferents being manipulated. As the present findings cannot be extrapolated to other test conditions, additional research would be needed to investigate the specific contribution of finger muscle afferents during LT to postural tasks in more stable conditions (e.g., with eyes open and standing on rigid surfaces).

Based on the results of the present study, it can be understood that the sensory information generated by the muscle spindles of the flexor muscle of the third finger of the hand during LT experiments was too small and/or not entirely accurate in terms of the position and movement of the fingertip to enhance postural stabilization associated with LT. However, this does not mean that this noisy or distorted sensory input would not be useful in more natural conditions of LT (i.e, with less restricted hand and arm postures). In such cases, its noisy or distorted representation could still be integrated (e.g., correlated) with the sensory inflow from a population of muscle spindles located at several muscles in the hand, arm, and torso, thereby contributing to an overall picture of each segment’s position and movement.

## Conclusion

The results showed that the removal of sensory input from the third finger flexor muscle did not lead to increased postural oscillations, i.e., muscle afferent inputs from the touching finger did not influence the beneficial effects of LT. These results suggest that the non-spindle afferents of the fingertip provide the physiologically relevant feedback from the touching finger to decrease postural sway during LT.

## Data Availability

All datasets generated for this study are included in the manuscript and/or the supplementary files.

## Ethics Statement

This study was carried out in accordance with the recommendations of the Human Ethics Committee of the Institute of Psychology at the University of São Paulo with written informed consent from all subjects. All subjects gave written informed consent in accordance with the Declaration of Helsinki. The protocol was approved by the Human Ethics Committee of the Institute of Psychology at the University of São Paulo.

## Author Contributions

AK and FM conceptualized the study. CS acquired and analyzed the data. CS prepared figures for the manuscript. CS, AK, and FM wrote and edited the manuscript. AK supervised the study.

### Conflict of Interest Statement

The authors declare that the research was conducted in the absence of any commercial or financial relationships that could be construed as a potential conflict of interest.

## Footnotes

^1^The simple main effects provided by *post hoc* pairwise comparisons are not possible through the standard SPSS dialog box, i.e., the software performs the pairwise comparisons for each of two factors, but it does not compare separate factors against each other. To circumvent this limitation, the appropriate SPSS commands were entered manually using a language of commands (SPSS Syntax Editor) (Field, 2017).
